# Impact of maternal physical activity on outcome of assisted reproduction

**DOI:** 10.3389/fendo.2026.1893897

**Published:** 2026-07-16

**Authors:** James Geiger, Alexander Quaas, Philipp Quaas, Andreas Schötzau, Viola Heinzelmann-Schwarz, Ursula Gobrecht-Keller, Manuel Fischer

**Affiliations:** 1 Department of Reproductive Medicine and Gynecological Endocrinology, University Hospital Basel, Basel, Switzerland; 2 Shady Grove Fertility San Diego, Solana Beach, CA, United States; 3 Eudox, Statistical Analysis, Basel, Switzerland; 4 Department of Gynecology & Gynecological Oncology, University Hospital Basel, Basel, Switzerland

**Keywords:** assisted reproductive technology (ART), cumulative live birth rate, health, physical activity, pregnancy rate

## Abstract

**Introduction:**

The influence of physical activity on outcomes of assisted reproductive technology (ART) remains incompletely understood, with conflicting evidence regarding the impact of exercise intensity and frequency. This study aimed to evaluate the association between self-reported physical activity and reproductive outcomes in patients undergoing ART.

**Methods:**

This retrospective single-center cohort study included 2,576 patients undergoing 6,850 ART cycles with embryo transfer between 2000 and 2021. Patients were categorized according to self-reported weekly physical activity as no regular exercise (Group A), exercise 1–2 times per week (Group B), and exercise ≥3 times per week, including competitive athletes (Group C). The primary outcome was cumulative live birth rate per oocyte pick-up (OPU), including fresh and frozen embryo transfers within one year following OPU. Secondary outcomes included cumulative pregnancy rate, pregnancy rate per embryo transfer, live birth rate per transfer, miscarriage rate, and number of oocytes retrieved.

**Results:**

Patients exercising 1–2 times weekly demonstrated the most favorable reproductive outcomes. Pregnancy rate per embryo transfer was highest in Group B (34.8%) compared with Group A (32.5%) and Group C (29.7%, p<0.005). Similarly, live birth rate per transfer was higher in Group B (25.6%) than in Groups A (23.0%) and C (22.4%, p<0.05). Cumulative pregnancy rate per OPU was highest in Group B (58.7%) compared with Group A (52.5%) and Group C (49.5%, p<0.001). Cumulative live birth rate per OPU was also higher in Group B (43.1%) compared with Group A (37.2%) and Group C (37.1%, p<0.01).

**Discussion:**

Moderate physical activity was associated with more favorable ART outcomes, including higher cumulative pregnancy and live birth rates, compared with both no regular exercise and high levels of physical activity. These findings suggest that moderate exercise may represent a beneficial lifestyle factor for patients undergoing ART and may support individualized patient counseling.

## Introduction

Lifestyle habits can significantly influence fertility, both naturally and in the context of assisted reproductive technology (ART). Factors such as obesity, poor diet, and toxic habits, including smoking and excessive alcohol consumption, have been shown to impair reproductive potential (1–4). The impact of physical activity on ART outcomes, including *in vitro* fertilization (IVF) and intracytoplasmic sperm injection (ICSI), has been examined in several studies, yielding inconsistent results.

In general, physical activity reduces the risk of hypertension, diabetes, metabolic syndrome, and associated cardiovascular complications (5). Its benefits for overall health are well established; however, in the context of ART, its effect remains controversial. Moderate physical activity appears to have a positive influence on certain reproductive outcomes. Systematic reviews and meta-analyses by Kakargia et al. and Rao et al. reported that physical activity was associated with significantly improved clinical pregnancy rates (CPR) and, in some studies, even improved live birth rates (LBR) following ART compared with inactivity (6, 7). Nevertheless, the evidence is inconsistent. Sõritsa et al. found that while higher physical activity and lower sedentary behavior were associated with improved controlled ovarian stimulation outcomes, they did not significantly affect implantation, pregnancy, or live birth rates (8). The largest prospective study to date, conducted by Morris et al., demonstrated reduced IVF success rates among physically active women (9). This is in line with the findings of Ricci et al., who concluded that maintaining moderate levels of physical activity may be beneficial, whereas high levels were not associated with improved outcomes (10). The very recently published study by Jacobs et al. was one of the first studies using wearable health trackers to examine *in vitro* fertilization outcomes and showed that physical activity and stress did not influence the pregnancy rate in programmed hormone replacement therapy (HRT) frozen embryo transfer cycles (11).

The intensity and type of physical activity also appear to be relevant, possibly more so than the overall amount. Prémusz et al. demonstrated a positive association between recreational physical activity and both the number of oocytes retrieved and the number of embryos developed (12).

As previously hypothesized by Kakargia et al., the J-curve pattern of physical activity in relation to cardiovascular and musculoskeletal health, described by O’Keefe et al., may also apply to patients undergoing ART (6, 13).

Because general health is a key factor for our patients, we have systematically recorded physical activity in all patients at our center for many years. Given the conflicting results in the current literature, we aimed to conduct a large-scale retrospective study to address this gap and provide more informed guidance for patients undergoing ART.

## Material and methods

### Study design

We conducted a retrospective single-center cohort study including all patients who underwent embryo transfer in an ART cycle from 2000 to 2021. The study evaluated the cumulative live birth rate per oocyte pick-up (OPU) based on the physical activity levels of the female patients. Fresh cycles with controlled ovarian stimulation and embryo transfer, as well as thaw cycles with embryo transfer, were each counted as one treatment episode (ART cycle).

### Inclusion and exclusion criteria

Patients were eligible if they underwent a completed ART cycle with embryo transfer (fresh or frozen) and had their physical activity assessed during the initial clinical evaluation. Patients who declined consent for research use of their data or had incomplete pregnancy outcome data were excluded. The study was conducted in accordance with applicable regulations. In accordance with Article 34 of the Swiss Human Research Act (HRA), the further use of biological material and health-related personal data for research without explicit consent was permitted under the conditions specified by the Act. Study population.

During the study period, couples planning to undergo ART underwent a routine check-up before starting IVF. Routine diagnostics included a basic gynecological examination (cycle monitoring), assessment of endocrine function, evaluation of physiological parameters, and patient anamnesis. During anamnesis, patients were asked about their weekly physical activity. Possible responses were: none (n = 2524), 1–2 times per week (n = 1676), 2–3 times per week (n = 939), 3–4 times per week (n = 481), 4–5 times per week (n = 18), 6 times per week (n = 6), occasionally (n = 853), daily (n = 340), or competitive sports (n = 13). Physical activity was only assessed prior to starting ART.

Patients were then divided into three groups. Group A included patients who did not engage in any sports. Group B included patients who exercised 1–2 times per week or occasionally. Group C included patients who exercised more than twice per week, daily, or competitively. Exercise was defined as a subset of physical activity characterized by planned, structured, and repetitive movements performed with the objective of improving or maintaining one or more components of physical fitness. Allocation to subgroups was verified by assessing heart rate as a reference marker for physical activity as regular exercise causes a reduction in resting heart rate. (**Table 1**) (14).

**Table 1:** Patient baseline characteristics of study cohorts and ART outcomes.

Parameter	Group A	Group B	Group C	Significance
ART cycles [n]	2524	2529	1797	
Patients [n]	985	936	655	
OPU cycles [n]	1561	1498	1074	
Age at OPU [y], mean	34.9	35.6	36.3	p<0.001^a^
Weight [kg], mean	66.0	64.1	63.9	p<0.001^a^
Heart rate [bpm], mean	74.4	72.5	69.7	p<0.001^a^
BMI [kg/m^2^], mean	24.2	22.7	22.6	p<0.001^a^
Obesity I° (30.0 – 34.9 kg/m^2^) [%]	9.9	3.0	1.2	p<0.001^b^
Obesity II° (35.0 – 39.9 kg/m^2^) [%]	3.0	0.9	0.6	p<0.001^b^
Obesity III° (≥ 40.0 kg/m^2^) [%]	4.6	0.8	0.3	p<0.002^b^
Smoker [%]	19.5	12.3	11.7	p<0.005^b^
Oocytes per OPU [n], mean	10.6	11.2	11.1	p<0.005^a^
Decreased ovarian reserve [n]/[%]	342/13.5	398/15.7	226/12.7	p<0.02^b^
Endometriosis [n]/[%]	160/6.3	197/7.7	126/7.1	n.s.^b^
Hypogonadotropic hypogonadism [n]/[%]	39/1.5	39/1.5	49/2.7	p<0.01^b^
PCOS [n]/[%]	182/7.2	129/5.1	125/7.0	p<0.005^b^
Recurrent miscarriage [n]/[%]	29/1.1	35/1.4	11/0.6	n.s.^b^
Thyroid disorder [n]/[%]	133/5.2	79/3.1	78/4.4	p<0.001^b^
Tubal pathology [n]/[%]	467/18.5	281/11.1	212/11.9	p<0.001^b^
Uterine fibroids [n]/[%]	99/3.9	61/2.4	38/2.1	p<0.001^b^
Pregnancy rate per ET [%]	32.5	34.8	29.7	p<0.005^b^
Ectopic pregnancy [n]	14	17	13	n.s.
Miscarriage per pregnancy [%]	29.1	26.5	24.6	n.s.
LBR per ET [%]	23.0	25.6	22.4	p<0.05^b^
Twin rate per live birth [%]	15.3	11.7	15.9	n.s.
Triplet rate per live birth [%]	2.1	2.0	1.2	n.s.
Birth weight [g], mean	3142	3096	3089	n.s.

(A: no exercising group, B: moderate exercising group, C: intensive exercising group).

^a^ ANOVA one way factorial analysis.

^b^ Chi-squared test.

### Stimulation protocols

Controlled ovarian stimulation was performed using either standard agonist or antagonist protocols, with administered medications and protocols varying over the course of the study. Until September 2017, all cryo-cycles were performed with frozen-thawed zygotes, as embryo cryopreservation beyond the zygote stage was not permitted in Switzerland until that time. Thereafter, clinical practice progressively shifted toward increased use of frozen blastocyte transfers.

Pregnancy follow-up was performed by reproductive specialists at the clinic. All ART treatments and resulting pregnancy outcomes were documented in a standardized manner in an electronic patient documentation system and reported to the national Swiss register of reproductive medicine (FIVNAT).

### Study outcome

The primary outcome was cumulative live birth rate, calculated as the total number of live births from one stimulation cycle and all consecutive cryo-cycles associated with the initial OPU within one year following a given oocyte retrieval. Secondary outcomes included the number of oocytes retrieved per OPU, biochemical pregnancy rate per transfer, miscarriage rate per pregnancy, live birth rate per transfer, and cumulative pregnancy rate per OPU.

Follow-up for cumulative birth and pregnancy rates extended one year after the last ART cycle in 2021.

### Statistical analysis

Descriptive statistical comparisons were performed by ANOVA one-way factorial analysis for quantitative data and chi-squared test for categorical data.

In case of cumulative pregnancy rate and live birth rate, nonlinear ordinal logistic regression (Proportional odds model: PO) was used to calculate odds ratios, using age, BMI, number of retrieved oocytes per oocyte pick-up, heart frequency, smoking behavior and physical activity as key predictors. Because the distribution of the three cohorts was not entirely homogeneous over the study period, with a higher proportion of the no-exercise group in the years before 2006 (A: 48%, p<0.001), we performed an additional sensitivity analysis by introducing treatment era as a categorical covariate in the logistic regression models for both cumulative pregnancy rate and cumulative live birth rate. The study period was divided into four treatment eras (2000–2005, 2006–2010, 2011–2015, and 2016–2021). Adjustment for treatment era did not materially alter the observed associations between physical activity and neither pregnancy rate nor cumulative live birth rate, indicating that the inhomogeneous distribution did not impact the primary outcome.

The number of retrieved oocytes was included in the primary model, as it represents a key biological determinant of cumulative reproductive potential. However, because a proportion of retrieved oocytes was never utilized for reproductive attempts following completion of family planning, the total number of oocytes may overestimate the clinically relevant reproductive potential. Therefore, we performed a sensitivity analysis by replacing the number of retrieved oocytes with the number of embryo transfers per oocyte pick-up, which reflects only those oocytes ultimately used for reproductive attempts. This analysis was conducted to assess the robustness of our findings.

In case of miscarriage rate, standard binary logistic regression was used. The PO model was chosen, because it is a natural generalization of the Wilcoxon-Test and has a simple interpretation as odds ratios (OR) – as described in the literature (15). Nonlinear modeling was used for Age, BMI, heartbeat rate as a three-knot restricted cubic spline.

Non-linear age dependency interpolation was adjusted for BMI (22.0) kg/m^2^, one ART cycle per oocyte pick-up, non-smoking behavior and heartbeat rate (72 bpm) and was graphically displayed. No interactions were included in the models.

Odds ratios of continuous or ordinal predictors were presented as the ratio of the odds increasing the predictor from the 25^th^ to the 75^th^ quartile representing a meaningful range. Odds ratios were presented with 95% confidence interval (CI) and corresponding p-values. A p-value of p<0.05 was considered as statistically significant.

Data collection processing and cohort evaluation was conducted using Microsoft Excel (Microsoft Corp., Redmond, WA, USA), R statistical software (R Foundation for Statistical Computing, Vienna, Austria) and SPSS (v28.0.1.0, IBM Corporation, USA).

### Ethics committee approval

Ethical approval was obtained by the institutional review board (Ethics Committee Northwest and Central Switzerland/Project-ID 2023-01866).

## Results

During the study period, 2576 patients underwent 6850 ART treatments with embryo transfer. Based on their weekly physical activity levels, they were distributed into three groups. Distribution was verified by mean heart rate. Group A included 2524 ART cycles of patients who did not engage in any sporting activity. Group B included 2529 ART cycles of patients who exercised one or two times per week. Group C included 1797 ART cycles of patients who exercised more than 2 times per week (**Supplementary Figure 1**). Significant differences were observed in the physiological baseline characteristics of the three groups, as expected for cohorts with varying levels of physical fitness (**Table 1**).

The mean age of the three patient groups increased with higher levels of physical activity (A: 34.9 years, B: 35.6 years, C: 36.3 years, p<0.001), while body weight (A: 66.0 kg, B: 64.1 kg, C: 63.9 kg, p<0.001), heartbeat rate (A: 74.4 bpm, B: 72.5 bpm, C: 69.7 bpm, p<0.001), BMI (A: 24.2 kg/m², B: 22.7 kg/m², C: 22.6 kg/m², p<0.001) and smoking behavior (A: 19.5% smokers, B: 12.3% smokers, C: 11.7% smokers, p<0.001) were inversely correlated with the level of weekly physical activity.

Fewer oocytes were collected per OPU in patients who did not exercise (A: 10.6 oocytes per OPU, B: 11.2 oocytes per OPU, C: 11.1 oocytes per OPU, p<0.005), despite the no-exercise group being younger and thus being expected to have an increased ovarian reserve. To evaluate the impact on the oocyte yield per OPU we performed a linear regression analysis (corrected R^2^ 0.094, ANOVA p<0.001) confounding for age (Beta -0.305, 95% CI -0.471 to -0.385, p<0.001), physical activity (Beta -0.065, 95% CI 0.272 to 0.773, p<0.001), Smoking (Beta -0.039, 95% CI -1.224 to -0.146, p=0.013) and BMI (Beta 0.001, 95% CI -0.045 to 0.046, p=0.979). Further factors might influence the amount of collected oocytes, which were not recorded in the course of the study.

We found that diagnosed pathologies (decreased ovarian reserve, endometriosis, hypogonadotropic hypogonadism, PCOS, recurrent miscarriage, thyroid disorder, tubal pathology, uterine fibroids) were not always evenly distributed between the three cohorts. To evaluate the impact of medical pathologies on MAR outcome we performed a binary logistic regression analysis and found that of all pathologies only hypogonadotropic hypogonadism (OR 1.73, p=0.004) and uterine fibroids (OR = 0.65, p=0.042) did impact live birth rate per embryo transfer (**Supplementary Table 1****).**

The pregnancy rate per embryo transfer was significantly different between groups B and C (A: 32.5%, B: 34.8%, C: 29.7%, p<0.05) (**Table 1**). Miscarriage rates per pregnancy did not differ significantly between groups when confounders were not considered (A: 29.1%, B: 26.5%, C: 24.6%, n.s.). Live birth rate per embryo transfer was significantly higher in group B (A: 23.0%, B: 25.6%, C: 22.4%, p<0.05), with no significant difference between groups A and C. No differences were observed in the rates of twins or triplets, or in the mean birth weight across the 1,630 assessed live births (**Table 1****).** Among all live births, 69 malformations and six postnatal complications were reported, and 10 infants died within one month after birth; no significant differences were found between the cohorts (data not shown). A significant impact of preimplantation genetic testing for aneuploidy (PGT-A) on pregnancy, live birth and miscarriage rate in this study is not to be expected since PGT-A was only allowed in Switzerland at the end of the study period. Thus, only five embryo transfers in total were performed with tested euploid embryos (2 embryo transfers allocating to group A, two embryo transfers allocating to group B and one embryo transfer allocating to group C).

Significant differences between the three groups were found for cumulative pregnancy rate and cumulative live birth rate, showing that group B achieved higher live birth outcomes. The cumulative pregnancy rate per OPU (A: 52.5%, B: 58.7%, C: 49.5%, p<0.001) and cumulative live birth rate per OPU (A: 37.2%, B: 43.1%, C: 37.1%, p<0.01) also differed significantly among the three groups (**Table 2**). Considering baseline characteristics, group B displayed intermediate confounding factors, such as age and BMI, suggesting that the observed effects were not solely attributable to these parameters.

**Table 2:** Patient baseline characteristics and cumulative ART outcomes.

Parameter	Group A	Group B	Group C	Significance
OPU cycles [n]	1561	1498	1074	
Age at OPU [y]	35.3	35.9	36.5	p<0.001^a^
BMI [kg/m^2^]	24.2	22.7	22.7	p<0.001^a^
Smoker [%]	20.5	12.1	12.0	p<0.001^b^
Heart frequency [bpm]	74.4	72.4	69.9	p<0.001^a^
Number of ET per OPU [n]	1.61	1.69	1.67	n.s.
Cumulative pregnancy rate per OPU [%]	52.5	58.7	49.5	p<0.001^a^
Cumulative LBR per OPU [%]	37.2	43.1	37.1	p<0.01^a^

(A: no exercising group, B: moderate exercising group, C: intensive exercising group).

^a^ ANOVA one way factorial analysis.

^b^ Chi-squared test.

Ordinal logistic regression analysis was performed to evaluate the influence of potential confounders on cumulative pregnancy and cumulative live birth rate per OPU. Standard logistic regression was used for miscarriage rate per pregnancy, including direct comparisons between all three groups and adjustment for age, BMI, smoking status, and number of ART cycles per OPU as well as physical activity category distribution across the study period.

**Figure 1:**
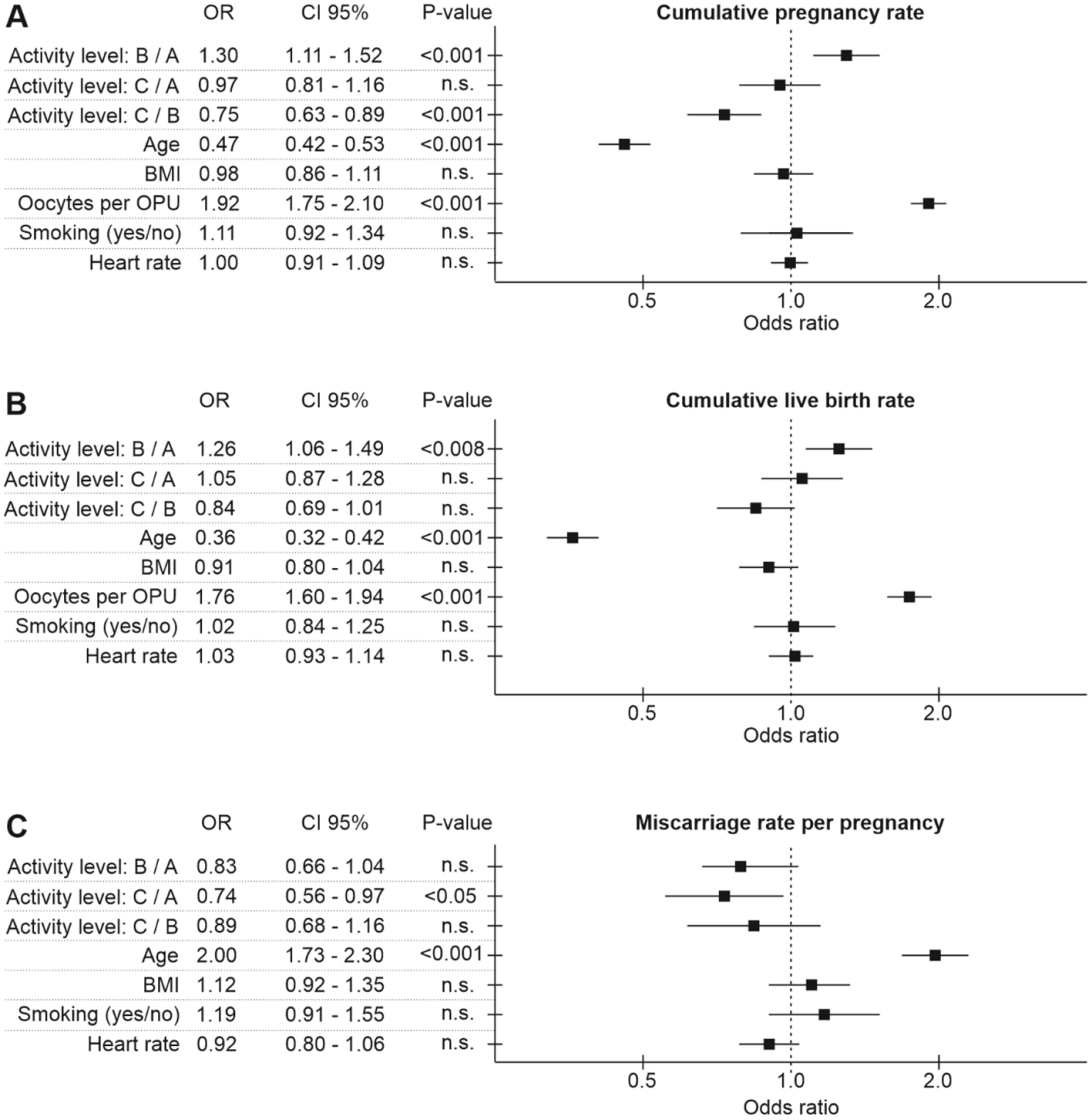
Influence of physical activity on outcome of ART cycles. **(A)** Impact on cumulative pregnancy rate of the level of physical activity, age, BMI, number oocytes per OPU and smoking behavior. Results were adjusted for the treatment era in which the OPU was performed (data not shown). **(B)** Impact on cumulative live birth rate of the level of physical activity, age, BMI, number of oocytes per OPU and smoking behavior. Results were adjusted for the treatment era in which the OPU was performed (data not shown). **(C)** Impact on miscarriage rate per pregnancy of the level of physical activity, age, BMI and smoking behavior.

Patient age had a strong negative impact on the cumulative pregnancy rate (cumulative pregnancy rate: OR 0.47, p<0.001), the cumulative live birth rate (cumulative LBR: OR 0.36, p<0.001) and increased miscarriage risk (Miscarriage: OR 2.00, p<0.001) (**Figures 1A–C**). Conversely, an increasing number of oocytes retrieved per OPU had a positive effect on cumulative pregnancy rate (OR 1.92, p<0.001) and cumulative live birth rate (OR 1.76, p<0. 001). No significant effects were observed for BMI or smoking status.

Moderate exercise (group B) was associated with improved outcomes. The odds ratio for cumulative pregnancy rate was higher in group B compared to group A (OR 1.30, p<0.001) and lower in group C compared to group B (OR 0.75, p<0.001), with no significant difference between groups C and A (OR 0.97, n.s.) (**Figure 1A**). For cumulative live birth rate, group B had higher odds compared to group A (OR 1.26, p<0.008), while no significant differences were observed between groups C and B (OR 0.84, n.s.) or groups C and A (OR 1.05, n.s.) (**Figure 1B**).

**Figure 2:**
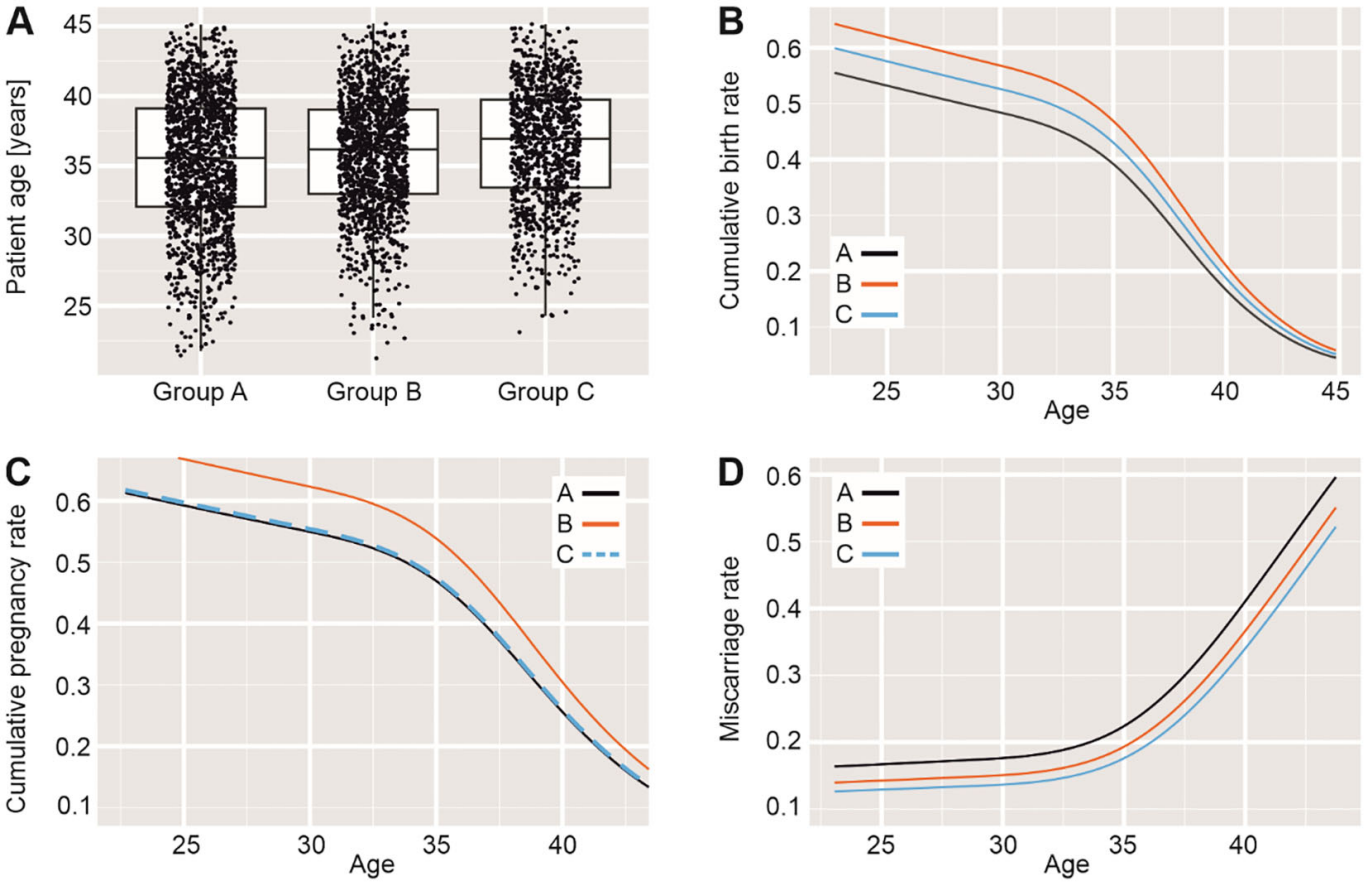
Distribution and correlation of age with no exercising group *(Group A)*, moderate exercising group *(Group B)* and intensive exercising group *(Group C).* **(A)** Age distribution of no exercising group, moderate exercising group and intensive exercising group – Boxplot indicating median and quartile. **(B)** Nonlinear dependence of cumulative pregnancy rate on patient age for no exercising group, moderate exercising group and intensive exercising group – adjusted for a BMI of 22, one ART cycle per OPU, non-smoking behavior and hear beat of 72 bpm. **(C)** Nonlinear dependence of cumulative live birth rate on patient age for no exercising group, moderate exercising group and intensive exercising group – adjusted for a BMI of 22, one ART cycle per OPU, non-smoking behavior and hear beat of 72 bpm. **(D)** Nonlinear dependence of miscarriage risk on patient age for no exercising group, moderate exercising group and intensive exercising group – adjusted for a BMI of 22 kg/m^2^, one ART cycle per OPU, non-smoking behavior and heartbeat of 72 bpm.

In the sensitivity analysis, replacing the number of retrieved oocytes with the number of embryo transfers per oocyte pick-up resulted in only minor changes in the effect estimates (absolute differences in ORs ranging from 3% to 10%). The direction, magnitude, and statistical significance of the associations between physical activity groups and cumulative live birth rate remained unchanged, demonstrating that the findings were robust to the choice of adjustment variable. The probability of a miscarriage was significantly reduced for group C compared to group A (miscarriage rate C/A: OR 0.74, p<0.05) (**Figure 1C**). No significant impact on odds ratio for miscarriage rate could be found when comparing group B to group A (miscarriage rate B/A: OR 0.83, n.s.) or comparing group C to group B (miscarriage rate C/B: OR 0.89, n.s.). It can be concluded that the chance for a miscarriage is lowest for patients of group C, exhibiting the highest level of physical exercise. This is despite the fact, that the mean age of group C was higher than the age of the other groups (**Figure 2A**).

Interpolation of cumulative pregnancy rate, cumulative live birth rate and miscarriage risk in dependency of patient age did show altered ART outcomes depending on physical activity of the cohorts, when adjusted for BMI, ART cycle number per OPU, smoking status, and heart rate (**Figure 2B-D**).

## Discussion

ART is a cost-intensive procedure that often imposes physical, emotional, and financial burdens on patients. Given the high drop-out rates following unsuccessful *in vitro* fertilization (IVF) cycles —ranging from 10% to over 60% — it is crucial to optimize concomitant factors to improve outcomes (16–18). Potential modifiable elements independent of medication protocols are lifestyle factors such as physical activity. Concerning the conflicting evidence in current literature regarding the effect of physical activity on ART outcomes, we conducted a retrospective study based on a large sample size of 2576 patients undergoing 6850 ART cycles.

A key factor limiting comparability between studies is the heterogeneity of groups with respect to physical activity levels and the lack of standardized definitions. In accordance with the literature, we defined three groups based on exercise frequency: none, moderate, and intense physical activity. We observed an inverse correlation between body weight, BMI, and resting heart rate with weekly physical activity (19). Previous studies have shown that regular aerobic exercise decreases resting heart rate and modestly reduces BMI, contributing to overall improvement of cardiovascular health and fitness. These benefits are evident even in the absence of significant weight loss, highlighting the importance of exercise for health beyond weight management alone (20–22).

To our knowledge, this study represents the largest sample size reported in the literature. We found that moderate physical activity showed significantly higher live birth rate per embryo transfer (**Table 1**: A 23.0%, B 25.6%, C 22.4%), as well as cumulative pregnancy rate (**Table 2**: A 52.5%, B 58.7%, C 49.5%) and cumulative live birth rate (**Table 2**: A 37.2%, B 43.1%, C 37.1%). These findings are consistent with a meta-analysis by Rao et al., which included eight studies and 3683 couples (7). In their subgroup analysis of studies controlled for age and BMI, the combined results showed an increase in LBR among women undertaking regular exercise (OR = 1.63, 95% CI 1.03-2.58, I^2^ = 0) with an intensity of >2.5 hours per week, excluding the prospective study by Morris et al. Notably, their scientific analysis showed no effect on LBR for regular exercise and even a decrease in LBR among women who reported engaging in more intense exercise (≥4 hours per week) 1–9 years in advance of their first IVF cycle (OR 0.6, CI 0.4-0.8). Interestingly, this negative impact was not observed in patients exercising ≥4 hours per week for more than 10 years, where exercise did not affect their IVF outcomes (9).

Consistent with these findings, our study demonstrated that moderate exercise increased cumulative pregnancy rates compared to no exercise (**Figure 1A**: OR 1.30, CI 1.11–1.52, p<0.001), whereas extensive exercise was associated with lower cumulative pregnancy rates compared to moderate exercise (**Figure 1A**: OR 0.75, CI 0.63–0.89, p<0.001).

Cumulative pregnancy rate and cumulative live birth rate were defined as the occurrence of one or more pregnancies or live births per oocyte retrieval, including the stimulated cycle and all subsequent cryo-cycles. The effect of maternal activity prior to an ART was assessed in a longitudinal study by Soritsa et al. (8). In general, a higher oocyte yield is favorable in ART by increasing the utilizable embryo number and therefore resulting in a higher cumulative LBR (23). However, the negative impact of physical inactivity on the primary outcomes can not only be explained by the significantly lower oocyte retrieval rates observed in inactive patients compared to those exercising regularly, since the results were adjusted for number of oocytes per OPU.

Elevated BMI, particularly in overweight and obese women, has been associated with an increased risk of miscarriage following IVF (24). In our study, BMI was not a significant factor for cumulative pregnancy rate, cumulative live birth rate, or miscarriage rate (**Figure 1A-C****).** However, the mean BMI across all study groups was below the obese range, and the relative similarity in BMI distribution may have limited the ability of this study to detect an effect of BMI on outcomes.

Notably, patients with high levels of physical activity exhibited a significantly decreased miscarriage rate per embryo transfer compared to inactive patients, after adjustment for other confounders (miscarriage rate C/A: OR 0.74, CI 0.56-0.97, p<0.05). This may indicate that high levels of physical activity is not synonymous with high levels of physical stress, which have been shown to have a negative impact on the miscarriage rate (25, 26). Obesity is associated with higher miscarriage rates (27, 28). Although miscarriage was only significantly reduced for patients exercising extensively, a trend towards lower miscarriage rates was also noticed for patients exercising at lower frequency (**Figure 1C**). This could potentially challenge the common belief that most miscarriages occur solely due to embryonic aneuploidy, which increases with maternal age. Our data indicates that physical activity may influence miscarriage risk, with higher activity levels being associated with a potentially more favorable risk profile. It remains unclear whether this effect is due to physical activity itself or to its positive effects on endocrine function and fertility-related health.

This study has several limitations. As a retrospective study, it is subject to inherent biases. Patient groups were categorized based on self-reported exercise frequency. Consequently, recall bias and social desirability bias may have influenced the assessment of physical activity, potentially leading to exposure misclassification (29). While we attempted to control potential confounding variables such as age, BMI, or smoking habits as much as possible, there is a residual risk of bias due to the retrospective nature of the study. The duration, intensity, and type of exercise were not adequately captured, and it was not possible to differentiate between strength and endurance activities, which may influence outcomes, as excessive cardiovascular exercise has been reported to negatively impact IVF results (9). Physical activity was assessed only prior to ART initiation and not during ovarian stimulation, embryo transfer, or early pregnancy. As physical activity may change substantially during these critical treatment phases due to physical discomfort, medical advice, or concerns regarding implantation, we cannot exclude exposure misclassification (8). In addition, increased physical activity in women may be associated with increased physical activity in their male partners, which could influence semen quality and sperm epigenetics (30). Therefore, differences in male fertility between the three cohorts might introduce a bias in ART outcomes of this study. Stimulation protocols evolved over the observation period, with an increase in short antagonist protocols in recent years. Until September 2017, all cryo-cycles were performed with frozen-thawed zygotes, as embryo cryopreservation was not permitted in Switzerland. Subsequent changes in Swiss reproductive law allowed single blastocyst transfers after 2017. However, these changes in protocols and legislative were independent of physical activity and are thus not expected to have an influence on the study outcomes as all patient cohorts were influenced equally by these factors.

In summary, our data did show that patients undergoing moderate physical exercise exhibit higher pregnancy and live birth rates, while patients with higher levels of physical exercise have a decreased miscarriage risk, independent of age and other baseline confounders.

In conclusion, moderate physical activity prior to ART is associated with higher pregnancy and cumulative live birth rates, while higher exercise levels are associated with the lowest miscarriage risk, independent of age and other baseline confounders. Larger prospective studies using objective measures of physical activity are warranted. Based on our findings and known general health benefits, moderate physical activity may be recommended for patients undergoing ART.

## Data Availability

The raw data supporting the conclusions of this article will be made available by the authors, without undue reservation.

## Supplementary material

Supplementary Figure 1 Study flow chart of the recruitment process. One ART cycle consists of either one fresh cycle with controlled ovarian stimulation followed by embryo transfer, or one cryo-cycle of frozen zygotes or blastocysts followed by embryo transfer. The patient population was divided into three groups based on their level of physical activity.

Supplementary Table 1 Binary logistic regression analysis of impact of medical diagnoses on live birth rate per embryo transfer.

Supplementary Table 2 Distribution of fresh cycles allocated to the three study groups for the whole study period and for four separate time subgroups of the study period.

Supplementary Table 3 Influence of physical activity on cumulative pregnancy rate by indicated co-variables, assessed by ordinal regression analysis.

Supplementary Table 4 Influence of physical activity on cumulative live birth rate by indicated co-variables, assessed by ordinal regression analysis.

## References

[1] Penzias A Azziz R Bendikson K Falcone T Hansen K Hill M . Obesity and reproduction: a committee opinion. Fertil Steril. (2021) 116:1266–85. doi: 10.1016/j.fertnstert.2021.08.018 34583840

[2] Panth N Gavarkovs A Tamez M Mattei J . The influence of diet on fertility and the implications for public health nutrition in the United States. Front Public Heal. (2018) 6:211. doi: 10.3389/fpubh.2018.00211 30109221 PMC6079277

[3] Augood C Duckitt K Templeton AA . Smoking and female infertility: a systematic review and meta-analysis. Hum Reprod. (1998) 13:1532–9. doi: 10.1093/humrep/13.6.1532 9688387

[4] Lyngsø J Ramlau-Hansen CH Bay B Ingerslev HJ Strandberg-Larsen K Kesmodel US . Low-to-moderate alcohol consumption and success in fertility treatment: a Danish cohort study. Hum Reprod. (2019) 34:1334–44. doi: 10.1093/humrep/dez050 31241750

[5] Patterson R McNamara E Tainio M de Sá TH Smith AD Sharp SJ . Sedentary behaviour and risk of all-cause, cardiovascular and cancer mortality, and incident type 2 diabetes: a systematic review and dose response meta-analysis. Eur J Epidemiol. (2018) 33:811–29. doi: 10.1007/s10654-018-0380-1 29589226 PMC6133005

[6] Kakargia E Mamalakis E Frountzas M Anagnostou E Siristatidis C . The role of maternal physical activity on *in vitro* fertilization outcomes: a systematic review and meta-analysis. Arch Gynecol Obstet. (2023) 307:1667–76. doi: 10.1007/s00404-022-06606-0 35596747

[7] Rao M Zeng Z Tang L . Maternal physical activity before IVF/ICSI cycles improves clinical pregnancy rate and live birth rate: a systematic review and meta-analysis. Reprod Biol Endocrinol. (2018) 16:11. doi: 10.1186/s12958-018-0328-z 29415732 PMC5803901

[8] Sõritsa D Mäestu E Nuut M Mäestu J Migueles JH Läänelaid S . Maternal physical activity and sedentary behaviour before and during *in vitro* fertilization treatment: a longitudinal study exploring the associations with controlled ovarian stimulation and pregnancy outcomes. J Assist Reprod Genet. (2020) 37:1869–81. doi: 10.1007/s10815-020-01864-w PMC7468008 32578030

[9] Morris SN Missmer SA Cramer DW Powers RD McShane PM Hornstein MD . Effects of lifetime exercise on the outcome of *in vitro* fertilization. Obstet Gynecol. (2006) 108:938–45. doi: 10.1097/01.aog.0000235704.45652.0b 17012457

[10] Ricci E Noli S Ferrari S La Vecchia I De Cosmi V Castiglioni M . Pretreatment maternal lifestyle and outcomes of assisted reproduction: an Italian cohort study. BMJ Open. (2020) 10:e038837. doi: 10.1136/bmjopen-2020-038837 33243794 PMC7692844

[11] Jacobs E Summers K Voorhis B Van DM . The impact of physical activity and stress on frozen embryo transfer cycles: the step and stress tracking to estimate pregnancy (SSTEP) trial. Fertil Steril. (2026) 125:401–10. doi: 10.1016/j.fertnstert.2025.08.039 40915598

[12] Prémusz V Makai A Perjés B Máté O Hock M Ács P . Multicausal analysis on psychosocial and lifestyle factors among patients undergoing assisted reproductive therapy - with special regard to self-reported and objective measures of pre-treatment habitual physical activity. BMC Public Health. (2021) 21:1480. doi: 10.1186/s12889-020-09522-7 PMC8063288 33892655

[13] O’Keefe EL Torres-Acosta N O’Keefe JH Lavie CJ . Training for longevity: the reverse J-curve for exercise. Mo Med. (2020) 117:355–61. PMC7431070 32848273

[14] Sandercock GRH Hardy-Shepherd D Nunan D Brodie D . The relationships between self-assessed habitual physical activity and non-invasive measures of cardiac autonomic modulation in young healthy volunteers. J Sports Sci. (2008) 26:1171–7. doi: 10.1080/02640410802004930 18608846

[15] Harrell FE . Regression Modeling Strategies. 2nd Editio. New York: Springer Cham (2015).

[16] Bensdorp AJ Tjon-Kon-Fat R Verhoeve H Koks C Hompes P Hoek A . Dropout rates in couples undergoing *in vitro* fertilization and intrauterine insemination. Eur J Obstet Gynecol Reprod Biol. (2016) 205:66–71. doi: 10.1016/j.ejogrb.2016.08.018 27567361

[17] Schröder AK Katalinic A Diedrich K Ludwig M . Cumulative pregnancy rates and drop-out rates in a German IVF programme: 4102 cycles in 2130 patients. Reprod BioMed Online. (2004) 8:600–6. doi: 10.1016/S1472-6483(10)61110-8 15151731

[18] Troude P Guibert J Bouyer J de La Rochebrochard E . Medical factors associated with early IVF discontinuation. Reprod BioMed Online. (2014) 28:321–9. doi: 10.1016/j.rbmo.2013.10.018 24461478

[19] Reimers AK Knapp G Reimers C-D . Effects of exercise on the resting heart rate : A systematic review and meta-analysis of interventional studies. (2018) 7(12):503. doi: 10.3390/jcm7120503 PMC6306777 30513777

[20] Höchsmann C Dorling JL Apolzan JW Johannsen NM Hsia DS Church TS . Effect of different doses of supervised aerobic exercise on heart rate recovery in inactive adults who are overweight or obese: results from E-MECHANIC. Eur J Appl Physiol. (2019) 119:2095–103. doi: 10.1007/s00421-019-04198-3 PMC6697220 31367909

[21] King NA Hopkins M Caudwell P Stubbs RJ Blundell JE . Beneficial effects of exercise: shifting the focus from body weight to other markers of health. Br J Sports Med. (2009) 43:924–7. doi: 10.1136/bjsm.2009.065557 19793728

[22] Marwick TH Hordern MD Miller T Chyun DA Bertoni AG Blumenthal RS . Exercise training for type 2 diabetes mellitus: impact on cardiovascular risk: a scientific statement from the American Heart Association. Circulation. (2009) 119:3244–62. doi: 10.1161/CIRCULATIONAHA.109.192521 19506108

[23] Venetis CA Tilia L Panlilio E Kan A . Is more better? A higher oocyte yield is independently associated with more day-3 euploid embryos after ICSI. Hum Reprod. (2019) 34:79–83. doi: 10.1093/humrep/dey342 30476100

[24] Rittenberg V Sobaleva S Ahmad A Oteng-Ntim E Bolton V Khalaf Y . Influence of BMI on risk of miscarriage after single blastocyst transfer. Hum Reprod. (2011) 26:2642–50. doi: 10.1093/humrep/der254 21813669

[25] Facchinetti F Matteo ML Artini GP Volpe A Genazzani AR . An increased vulnerability to stress is associated with a poor outcome of *in vitro* fertilization-embryo transfer treatment. Fertil Steril. (1997) 67:309–14. doi: 10.1016/s0015-0282(97)81916-4 9022608

[26] Geisler M Meaney S Waterstone J O’Donoghue K . Stress and the impact on the outcome of medically assisted reproduction. Eur J Obstet Gynecol Reprod Biol. (2020) 248:187–92. doi: 10.1016/j.ejogrb.2020.03.006 32240891

[27] Broughton DE Moley KH . Obesity and female infertility: potential mediators of obesity’s impact. Fertil Steril. (2017) 107:840–7. doi: 10.1016/j.fertnstert.2017.01.017 28292619

[28] Bellver J Rossal LP Bosch E Zúñiga A Corona JT Meléndez F . Obesity and the risk of spontaneous abortion after oocyte donation. Fertil Steril. (2003) 79:1136–40. doi: 10.1016/s0015-0282(03)00176-6 12738508

[29] Adams SA Matthews CE Ebbeling CB Moore CG Joan E Fulton J . The effect of social desirability and social approval on self-reports of physical activity. (2005) 161:389–98. doi: 10.1093/aje/kwi054 PMC2958515 15692083

[30] Donato F Rota M Ceretti E Viviana Viola GC Marullo M Zani D . Intensity and type of physical activity and semen quality in healthy young men. In: Fertil Steril, vol. 123. New York: Elsevier (2025). p. 88–96. Available online at: https://www.sciencedirect.com/science/article/pii/S001502822401954X?via%3Dihub. 39243273 10.1016/j.fertnstert.2024.08.323

