# Supplementary material for: Impact of maternal physical activity on outcome of assisted reproduction
Source: Front Endocrinol (Lausanne). 2026 Jul 16;17:1893897. doi: 10.3389/fendo.2026.1893897 (PMC13421417; doi:10.3389/fendo.2026.1893897)
Supplement: Supplementary Figure 1 — Study flow chart of the recruitment process. One ART cycle consists of either one fresh cycle with controlled ovarian stimulation followed by embryo transfer, or one cryo-cycle of frozen zygotes or blastocysts followed by embryo transfer. The patient population was divided into three groups based on their level of physical activity. [file Supplementaryfile1.pdf]

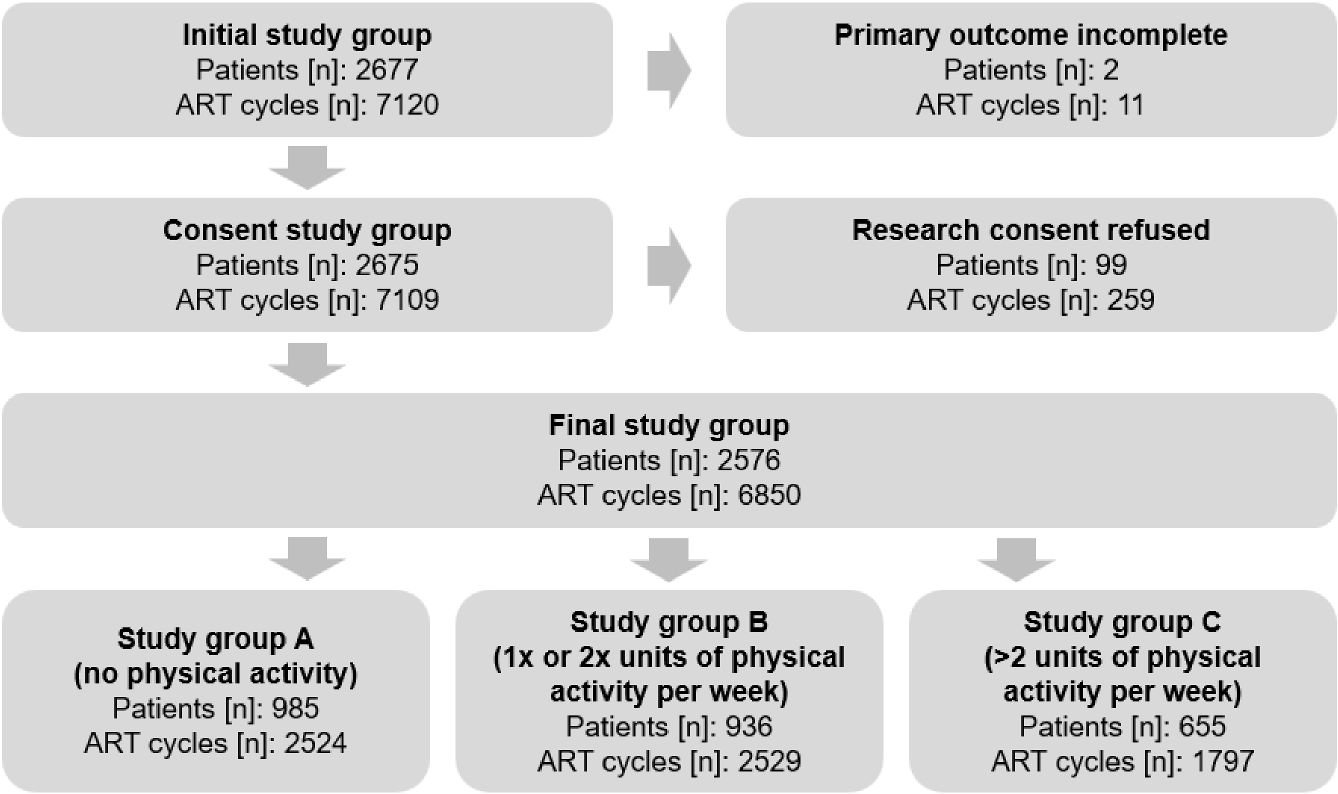

Supplementary Figure 1: Study flow chart of the recruitment process. One ART cycle consists of either one fresh cycle with controlled ovarian stimulation followed by embryo transfer, or one cryo-cycle of frozen zygotes or blastocysts followed by embryo transfer. The patient population was divided into three groups based on their level of physical activity.

Supplementary Table 1. Binary logistic regression analysis of impact of medical diagnoses on live birth rate per embryo transfer

Parameter	OR	95% CI	Significance
Age	0.91	0.90 to 0.92	p<0.001
Decreased ovarian reserve	0.86	0.72 to 1.02	n.s.
Endometriosis	1.07	0.86 to 1.33	n.s.
Hypogonadotropic hypogonadism	1.73	1.19 to 2.52	p=004
PCOS	1.10	0.88 to 1.38	n.s.
Recurrent miscarriage	1.22	0.71 to 2.10	n.s.
Thyroid disorder	0.90	0.68 to 1.19	n.s.
Tubal pathology	0.85	0.72 to 1.02	n.s.
Uterine fibroids	0.65	0.42 to 0.99	p=042

Supplementary Table 2. Distribution of fresh cycles allocated to the three study groups for the whole study period and for four separate time subgroups of the study period

Study Group	Total period	2000 - 2005	2006 - 2010	2011 - 2015	2016 - 2021	Significance
A	1561 (37.8%)	294 (51.1%)	442 (36.4%)	469 (35.8%)	356 (34.4%)	
B	1498 (36.2%)	149 (25.9%)	473 (38.9%)	496 (37.9%)	380 (36.8%)	
C	1074 (26.0%)	132 (23.0%)	300 (24.7%)	344 (26.3%)	298 (28.8%)	
Total fresh cycles	N=4133	N=575	N=1215	N=1309	N=1034	p<0.001

Supplementary Table 3. Influence of physical activity on cumulative pregnancy rate by indicated co-variables, assessed by ordinal regression analysis

Parameter	OR	95% CI	Significance
Smoking	1.11	0.92 to 1.34	n.s.
Heart rate	1.00	0.91 to 1.09	n.s.
Oocytes per OPU	1.92	1.75 to 2.10	p<0.001
BMI	0.98	0.86 to 1.11	n.s.
Age	0.47	0.42 to 0.53	p<0.001
Activity level C / B	0.75	0.63 to 0.89	p<0.001
Activity level C / A	0.97	0.81 to 1.16	n.s.
Activity level B / A	1.30	1.11 to 1.52	p<0.001
Time period 2000-2005 / 2011-2015	1.31	1.06 to 1.61	p=0.013
Time period 2006-2010 / 2011-2015	1.26	1.07 to 1.49	p=0.007
Time period 2016-2021 / 2010-2015	1.71	1.40 to 2.08	p<0.001

Supplementary Table 4. Influence of physical activity on cumulative live birth rate by indicated co-variables, assessed by ordinal regression analysis

Parameter	OR	95% CI	Significance
Smoking	1.02	0.84 to 1.25	n.s.
Heart rate	1.03	0.93 to 1.14	n.s.
Oocytes per OPU	1.76	1.60 to 1.94	p<0.001
BMI	0.91	0.80 to 1.04	n.s.
Age	0.36	0.32 to 0.42	p<0.001
Activity level C / B	0.84	0.69 to 1.01	n.s.

Activity level C / A	1.05	0.87 to 1.28	n.s.
Activity level B / A	1.26	1.06 to 1.49	p=0.008
Time period 2000-2005 / 2011-2015	1.35	1.08 to 1.69	p=0.009
Time period 2006-2010 / 2011-2015	1.47	1.23 to 1.76	p<0.001
Time period 2016-2021 / 2010-2015	1.87	1.51 to 2.32	p<0.001
